# A Response-Feedback-Based Strong PUF with Improved Strict Avalanche Criterion and Reliability

**DOI:** 10.3390/s24010093

**Published:** 2023-12-23

**Authors:** Baokui Zhu, Xiaowen Jiang, Kai Huang, Miao Yu

**Affiliations:** 1 College of Electrical Engineering, Zhejiang University, Hangzhou 310027, China; 2 School of Micro-Nano Electronics, Zhejiang University, Hangzhou 310027, China

**Keywords:** pysically unclonable function, response feedback, generalized strict avalanche criterion, reliability, machine learning, internet of things

## Abstract

Physical Unclonable Functions (PUFs) are significant in building lightweight Internet of Things (IoT) authentication protocols. However, PUFs are susceptible to attacks such as Machine-Learning(ML) modeling and statistical attacks. Researchers have conducted extensive research on the security of PUFs; however, existing PUFs do not always possess good statistical characteristics and few of them can achieve a balance between security and reliability. This article proposes a strong response-feedback PUF based on the Linear Feedback Shift Register (LFSR) and the Arbiter PUF (APUF). This structure not only resists existing ML modeling attacks but also exhibits good Strict Avalanche Criterion (SAC) and Generalized Strict Avalanche Criterion (GSAC). Additionally, we introduce a Two-Level Reliability Improvement (TLRI) method that achieves 95% reliability with less than 35% of the voting times and single-response generation cycles compared to the traditional pure majority voting method.

## 1. Introduction

The IoT is an interconnected network of various devices, such as smartwatches, medical equipment, cars, smart home devices, and sensors, among other embedded devices [1], which play a crucial role in data transmission. The interconnectivity of all things is the current trend, and according to predictions [2] the global IoT device total is expected to reach 75.4 billion by 2025. With the rapid development of the IoT, the security of authentication between devices is becoming increasingly important. For example, industrial wireless sensors need to upload the collected private data to the cloud server, resulting in a large amount of private data being exposed on the Internet [3]. The exposed data are easily accessible to attackers. However, authentication methods based on traditional cryptography (AES, Hash, etc.) consume significant hardware resources and are difficult to apply to resource-constrained IoT devices [4]. Moreover, this method requires storing keys, which can be obtained by attackers through physical attacks such as probing [5], posing a significant security risk. As a hardware primitive, PUF utilizes process variations during chip manufacturing to generate a unique device ID [6], and it has advantages such as being lightweight, easy to implement, and without the need for storage of responses. Therefore, it is suitable for authentication protocols in resource-constrained IoT environments [7,8]. PUF can be classified into weak PUF and strong PUF according to the number of the Challenge Response Pairs (CRPs). Weak PUF, such as SRAM PUF [4], has small CRPs space and is mainly used in traditional encryption systems to generate unique chip keys. Strong PUF has a response space that exponentially increases with the number of challenges. Based on this feature, strong PUF is more suitable for IoT device authentication than the weak PUF, because, after completing one authentication, the used CRPs can be discarded to prevent replay attacks. Arbiter PUF (APUF) [6] is one of the most widely applied strong PUFs, generating responses 0/1 via a race between the signals in two symmetric delay chains. However, due to the untrusted communication channel and the linear mathematical model (detailed description in Section 3), attackers can collect CRPs and launch attacks such as ML modeling [9]. What is more, because of its poor statistical characteristics, the APUF is vulnerable to statistical attacks [10]. Therefore, our security mainly focuses on resisting ML modeling and statistical attacks.

To resist ML modeling attacks, researchers have proposed solutions such as XORPUF [11], FFPUF [12], IPUF [13] and MPUF [14]. However, these methods have been modeled by different ML attacking methods [15] and cannot meet ideal statistical characteristics. Due to poor statistical characteristics, the security of many PUFs is threatened. Reference [16] proposes an approximate attack method. Through statistical analysis of CRPs in BR-PUF [17], it was found that when some challenge bits are fixed at 0/1, the output will bias towards 0/1 with a 90% probability. Based on this feature, they constructed statistical ML modeling attacks. SAC is an important criterion for measuring the security of encryption algorithms in cryptography [18]. This means that when any bit of the plaintext or key changes, nearly half of the bits in the ciphertext will flip. Researchers introduced this concept to evaluate the statistical characteristics of PUFs [19], which means that when any single bit of the challenge flips, the probability of the response flipping is 50%. Similarly, we can extend SAC to the GSAC, where the probability of response flipping is 50% no matter which bits are flipped. This concept is similar to the Propagation Criterion (PC) [20], but is stronger than that [10]. SAC and GSAC ensure the independence between two CRPs; that is, attackers cannot directly deduce the response of another challenge through one or several CRPs. LSPUF [21] and SPUF [22] can achieve good SAC through input conversion and output obfuscation. However, due to the flaws in its own structure and the emergence of various attack methods, its security cannot be guaranteed. On the one hand, the input structure of these two PUFs cannot resist reverse attacks and has been modeled [23]. On the other hand, reference [10] proposes an ACCA attack that utilizes the poor SAC of adjacent 2-bit challenge flipping in LSPUF to successfully predict the response with a high probability. Therefore, LSPUF cannot meet GSAC, and only meeting SAC cannot ensure resistance to statistical attacks. GSAC is important for PUF statistical security. Unfortunately, besides LSPUF and SPUF, MPUF [14] and [24] have only discussed SAC. FLAM-PUF [25] introduces response cyclic feedback, greatly enhancing the GSAC of PUF, but due to the influence of environmental noise, it is difficult for this structure to maintain good reliability, and the LFSR feedback polynomial after secondary confusion may not necessarily be the primitive polynomial, which will reduce the CRPs space. In response to the aforementioned security and reliability issues, this article mainly makes the following contributions:

- It proposes a response feedback PUF based on LFSR and APUF, which greatly enhances the nonlinearity and randomness of the structure and enhances its ability to resist ML modeling and GSAC through response cyclic feedback. At the same time, the structure will not be subjected to reverse attacks. The response feedback does not affect the feedback bits of LFSR, thus ensuring that the feedback polynomial of LFSR is the primitive polynomial and the PUF CRPs space is not affected.
- It proposes Two-Level Reliability Improvement (TLRI), which is based on traditional voting methods to enhance reliability. By incorporating a reliable bit screening mechanism, this method can reduce the number of voting and single-response generation cycles by more than 65% compared to pure voting methods when the reliability is improved to 95%.
- Through simulation, we have demonstrated for the first time that this structure has good GSAC; even in a worst-case scenario, its output flip probability only deviates from the ideal value (0.5) by approximately 0.05. At the same time, ML modeling attacks such as LR, DNN, and SVM have a prediction accuracy of no more than 60%.

The remaining pars of this paper are organized as follows: Section 2 introduces related work, including the application of LFSR in PUF, the current status of feedback-based PUF, and methods for improving PUF reliability. Section 3 presents the specific structures and mathematical models of LFSR and APUF. Section 4 provides the specific structure of the proposed PUF, analyzes the process of confusion and response generation, and conducts security and reliability analysis. Section 5 conducts reliability, GSAC, and resistance to ML attack capability analysis based on Python simulation. Finally, the conclusion is presented.

## 2. Related Works

LFSR is an important concept in the fields of integrated circuits and cryptography (detailed description in Section 3). In PUF applications, it is usually used as a challenge generator [26]. When a challenge is received, it generates a set of sub-challenges through shifting, thereby reducing the communication burden. From another perspective, the sub-challenge can be seen as a confusion of the original challenge. The shifting of the LFSR introduces nonlinearity between the two. The CRC PUF [27] was the first to utilize the above characteristics of the LFSR to enhance the PUF’s resistance to ML modeling attacks. However, under reverse attacks, once the feedback polynomial and initial seed are obtained, its output can be deduced, leading to the failure of its confusion. Therefore, the authors further enhanced security by dynamically changing the feedback coefficient, but they did not provide a detailed description of how to do that. In order to solve this problem, SR-PUF [28] uses the responses of n APUFs to dynamically update the feedback coefficients of the LFSR. The randomness of the APUF response ensures that the LFSR feedback coefficients are not obtained by attackers, thereby resisting reverse attacks. However, this not only brings unaffordable hardware consumption but also greatly decreases the reliability of the response. DCH [29] and DCT [30] use dynamic reconstruction of the PUF structure by the LFSR, where the LFSR state changes every clock cycle and is unrelated to the challenge, so attackers cannot obtain its real-time state. However, on the one hand, authentication protocols based on this PUF require the use of pattern matching, resulting in significant communication overhead. On the other hand, its underlying PUF is APUF, and according to [31] it cannot meet SAC and GSAC. In this article, while using LFSR for obfuscation, we introduce response feedback in the proposed structure that, even if the sequence of LFSR is obtained, the true challenge of PUF remains unknown.

Response feedback is an important way to enhance the nonlinearity of PUF, and FF-PUF [12] is the first structure to apply this method. The authors introduce a forward feedback branch in the APUF, using the APUF response from the intermediate stage as the challenge for the later stage, thereby introducing nonlinearity. Shah et al. [32] proposed the Rec-PUF, which feeds the response back to the challenge side to achieve multi-round confusion of the challenge. In the first round, the original challenge is directly input to the underlying PUF, the response is XORed with the challenge, and the result is used as the challenge of the PUF for the next round. The PUF response, after multiple rounds of confusion, serves as the final response. Different numbers of response feedback and iteration times will yield different results. Due to the randomness of PUF responses, the more feedback numbers and iterations, the stronger the introduced randomness and the stronger the resistance to ML modeling. However, in reality, PUF outputs are not 100% reliable due to noise from the variation of temperature and voltage [33]. As the number of feedback iterations increases, the introduced noise in the system also gradually increases. According to the results of Shah et al. [32], when the number of feedback iterations increases to 8, the reliability of the response output is less than 65%. Similarly, FLAM-PUF [25] introduces an LFSR between the challenge and the PUF and changes the feedback coefficients of the LFSR through two confusion stages, introducing nonlinearity and randomness. In the first stage, a single-bit response is fed back to a certain feedback coefficient of the LFSR, undergoing *n* − 1 cycles of confusion and collecting *n* − 1 bits of response. In the second stage, the *n* − 1 feedback coefficients of the LFSR are replaced by the collected responses in the first stage. Similar to Rec-PUF, multiple feedbacks introduce a large amount of noise, resulting in very low structural reliability. Additionally, due to the randomness of the PUF response, the feedback polynomial of the LFSR may not necessarily be a primitive polynomial, which will reduce the CRPs space and thus pose security risks.

Reliability is one of the most important characteristics of PUFs. Many strong PUFs incorporate numerous XOR and feedback operations to enhance the nonlinearity of the PUF and improve its resistance to ML modeling, but this reduces reliability [34]. ECC-based algorithms for post-processing responses, relying on helper data, are widely used [4]. However, the algorithm itself requires excessive area consumption and the helper data need to occupy non-volatile memory, resulting in significant hardware costs. Additionally, Majority Voting (MV) [35] is a common method for improving the reliability of strong PUFs. This method selects the most frequently occurring response through multiple evaluations of the PUF response, thereby filtering out noise. When introducing less noise, this method can achieve higher reliability with a reasonable number of votes [36]. However, when too much noise is introduced, the number of votes can become extremely large [37], resulting in a significant increase in response generation time. Reference [38] uses the 4-DFF or SR latch-based arbiter to construct a metastability detection circuit, selecting CRPs that do not exhibit metastability. However, this method leads to response imbalance and additional encoding overhead [39]. The Bit-Self-Test (BST) APUF [39] significantly improves reliability by constructing additional delay circuits to select responses with longer path delays. In this paper, a TLRI circuit is constructed based on BST and the MV method, greatly reducing the number of votes while improving the reliability of the PUF.

## 3. Grounded Theory

**Figure 1:**
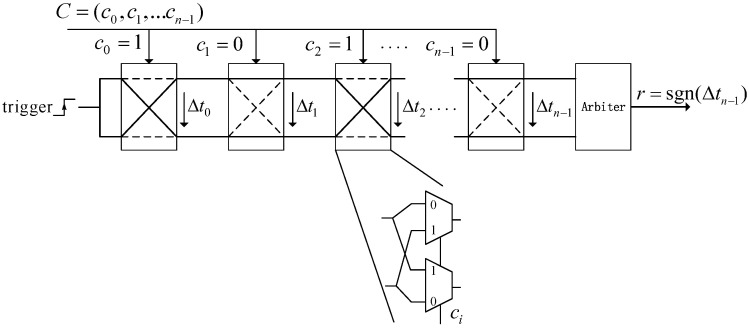
Structure of APUF.

APUF is a delay-based PUF composed of two symmetric multiplexer chains (*n* stages, upper and lower) and a final arbiter. The arbiter can be a Flip-Flop or latch. The same trigger signal propagates in parallel on two paths. Its input is the *n* bit challenge $C=(c_{0},c_{1},\ldots ,c_{n-1})$. The crossing or passing through of the path in each stage is determined by the challenge $c_{i}$. $\Delta t_{i}$ is the sum of delay difference up to the stage *i*, and $\Delta t_{n-1}$ is the delay difference of the whole APUF. The positive or negative value of $\Delta t_{n-1}$ determines whether the response *r* is 1 or 0. Its structure is shown in Figure 1.

**Figure 2:**
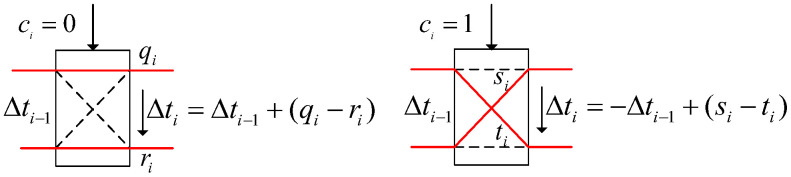
Delay difference.

The additive linear delay model [40,41] is commonly used as the mathematical model for APUF. The specific delay of each stage is shown in Figure 2. The difference in delay for two symmetrical paths in each stage with $c_{i}=0$ and $c_{i}=1$ are $d_{i}^{0}=q_{i}-r_{i}$ and $d_{i}^{1}=s_{i}-t_{i}$, respectively. Similarly, $\Delta t_{i}=\Delta t_{i-1}+d_{i}^{0}$ and $\Delta t_{i}=-\Delta t_{i-1}+d_{i}^{1}$, respectively.

Therefore, we can obtain $\Delta t_{i}=\left(1-2c_{i}\right)\Delta t_{i-1}+\left(1-c_{i}\right)d_{i}^{0}+c_{i}d_{i}^{1},\;i\geq 1$. Final delay difference $\Delta t_{n-1}$ can be expressed as

(1) $$\Delta t_{n-1}=\vec{\omega }\vec{\phi },$$

where $\vec{\omega }\;=\left(\omega_{0},\omega_{1},\ldots ,\omega_{n}\right)$ is the delay vector, determined by the delay difference of each stage. The delay difference originates from manufacturing process variations. Therefore, $\vec{\omega }$ should be unique for each APUF.

(2) $$\begin{array}{cc} \; & \omega_{0}=\left(d_{0}^{0}-d_{0}^{1}\right)/2\\ \; & \omega_{i}=\left(d_{i-1}^{0}+d_{i-1}^{1}+d_{i}^{0}-d_{i}^{1}\right)/2,\;i=1,2,\ldots ,n-1\\ \; & \omega_{n}=\left(d_{n-1}^{0}+d_{n-1}^{1}\right)/2 \end{array}$$

$\vec{\phi }\;={\left(\phi_{0},\phi_{1},\ldots ,\phi_{n-1},1\right)}^{T}$ is the feature vector, determined by challenge *C*:

(3) $$\phi_{l}=\prod_{i=l}^{n-1}(1-2C_{i}),\;l=0,1,\ldots ,n-1$$

For ease of calculation and analysis, when the response is 0, it is set to −1; the expression for APUF response is as follows: sgn(.) is the sign function, *r* is −1 if $\Delta t_{n-1}\leq 0$, else *r* is 1.

(4) $$r=\mathrm{sgn}\left(\Delta t_{n-1}\right)=\mathrm{sgn}\left(\vec{\omega }\;\vec{\phi }\;\right)$$

According to Equation (1), there is only a simple linear relationship between PUF response and $\vec{\omega }$, $\vec{\phi }$. Therefore, once the APUF mathematical model is known, the attacker can directly calculate $\vec{\phi }$ based on challenge *C* and feed it along with the responses into the machine learning model for training, thus quickly iterating to obtain the correct $\vec{\omega }$. However, if the attacker is unaware of the model, it can be inferred from Equation (3) that there is a non-linear relationship between challenge *C* and response. Therefore, the difficulty in modeling will greatly increase. According to the research of [24], taking the APUF additional delay model as known, artificial neural network modeling attacks can achieve a modeling success rate of 98.3% for APUF in only 2K CRPs in one minute. However, if the model is unknown, even if the training data reach 1 million its modeling accuracy is only 60.7%. Therefore, it is important to introduce nonlinearity between challenge and response. In this article, we achieved this through the shift and response feedback of LFSR.

**Figure 3:**
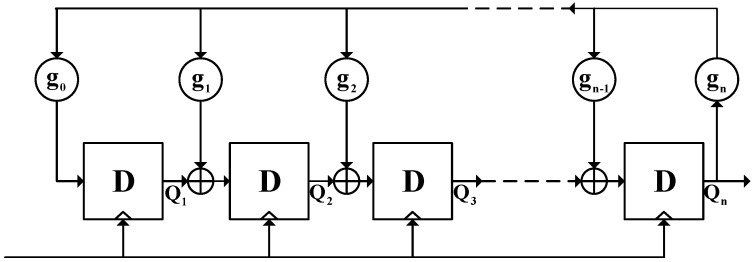
Galois LFSR.

LFSR consists of a set of registers and a set of logic gates, which shift the bits in the registers and perform XOR operations based on specific feedback polynomials to generate the output of the next clock cycle. The output sequence of LFSR depends on the initial state (seed) and feedback polynomial. When the initial state and feedback polynomial are determined, LFSR will generate a pseudo random output sequence according to a certain law. There are two types of LFSR structures, Fibonacci and Galois. This article only focuses on Galois LFSR, as shown in Figure 3.

In the above figure, $g_{i}$ is the feedback coefficient, taken as 0/1, where 1 means it exists in the feedback branch and 0 means it does not exist. The feedback polynomial relative to it is $f\left(x\right)=g_{n}x^{n}+g_{n-1}x^{n-1}+\ldots +g_{0}x^{0}$. The LFSR circuit, composed of n triggers, can generate an output sequence with a maximum period of $2^{n}-1$. The feedback polynomial corresponding to the maximum sequence is the primitive polynomial. To make the output sequence the maximum sequence, it is also necessary to ensure that the initial seed is not all zero.

## 4. Proposed PUF

### 4.1. Overview of Proposed PUF

**Figure 4:**
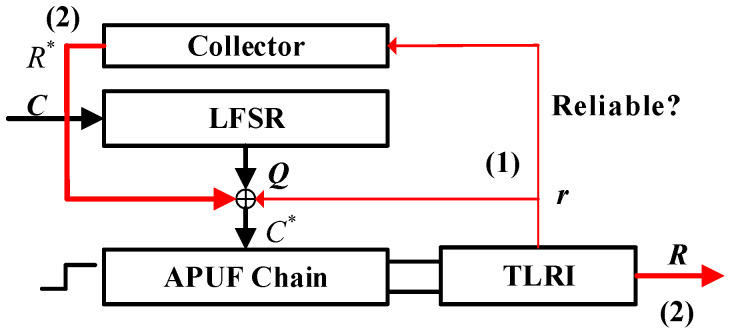
Structure of proposed PUF.

This paper proposes a PUF structure, as shown in Figure 4, consisting of an APUF chain, an LFSR with primitive polynomial, and TLRI. The APUF chain is composed of two symmetric multiplexer chains, obtained by removing the arbiter from the APUF in Figure 1. The collector temporarily stores the APUF response and can be implemented by n shift registers. TLRI is a response reliability detection and enhancement circuit, which plays a crucial role in the reliability of the final response. It mainly consists of response delay detection and MV circuits; its specific structure is analyzed in Section 4.3. In this figure, (1) and (2) mean stages 1 and 2. Stage 1 is the first confusion process that includes n shifts of LFSR and response feedback, introducing nonlinearity and randomness. In stage 2, the second confusion is introduced by XORing the data in the collector with the output sequence of LFSR, and the final response is generated. Thin red lines and lowercase letters represent the single response generated in each cycle in stage 1, while thick red lines and uppercase letters represent the data in the collector and the final response.

In stage 1, in every cycle the LFSR generates new output *Q* by shifting, and the APUF generates a response, which is then checked for reliability through TLRI. If the bit response has high reliability, it is fed back to the output of the LFSR and XORed with certain bits, with the confused result used as the challenge of the APUF for the next cycle, and the response is collected in a collector. After n shifts, the data in the collector are XORed with the output of the LFSR to serve as the challenge for the final response. According to [10], when the number of bit flips in the challenge is odd and the *n*-bit challenge is evenly divided, the probability of APUF response flipping is closest to 0.5. Therefore, we choose to XOR the output of the LFSR at the $\frac{n}{4}$, $\frac{n}{2}$, $\frac{3n}{4}$ positions with the feedback response. For a 64-bit APUF, the feedback bit is XORed with the 16th, 32nd, and 48th challenges. The detailed process of the response generation is as follows:

Initialization: Firstly, initialize the LFSR with the original challenge *C* and generate the first response with this original challenge from APUF. If the response is reliable enough, as checked by the TLRI, it is fed back to the output *Q* of the LFSR and XORed with $Q_{\frac{n}{4}}$, $Q_{\frac{n}{2}}$, $Q_{\frac{3n}{4}}$ for the next cycle to generate challenge $C^{*}$. The initial value of the feedback bit is 0.

**Figure 5:**
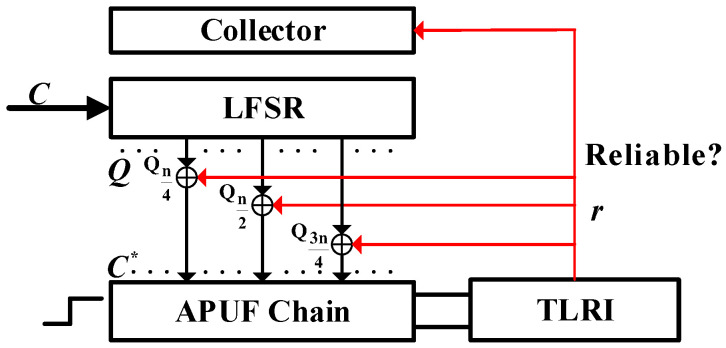
Stage 1 of proposed PUF.

Stage 1: This stage is an important phase for introducing system nonlinearity and randomness, which is significant for enhancing PUF’s resistance to ML modeling attacks and GSAC. In each cycle, LFSR produces the output *Q*, XORed with the feedback bit as the challenge of the APUF. Then, the APUF produces a response *r*. If the single response is sufficiently reliable, it is fed back to the challenge side, XORed with *Q*, and stored in the collector. The above steps are repeated n times, from the first cycle to the *n*th cycle. What is different is that when the response is unreliable, the reliable response from before will be XORed with *Q* as the feedback bit, but the collector collects 0. The initial value of the collector is n zeros, and the value after stage 1 will be determined by the number of reliable responses. For the sake of completeness and fluency in the analysis, Figure 5 briefly illustrates the feedback process of the response. The specific process refers to the TLRI structure in Section 4.3.

**Figure 6:**
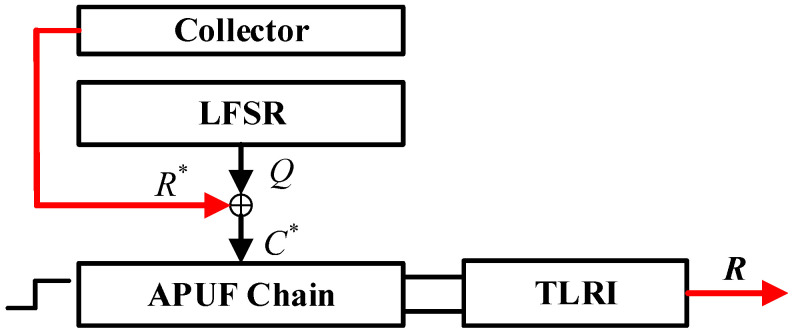
Stage 2 of proposed PUF.

Stage 2: After stage 1, starting from the (*n* + 1)th cycle, the *n*-bit data in the collector are XORed with the LFSR output *Q*, and no further feedback of responses occurs, as shown in Figure 6. There are two reasons for performing this operation. Firstly, continuous response feedback will continuously introduce noise, making it difficult to ensure the reliability of subsequent responses. Secondly, in order to ensure that the LFSR feedback polynomial is primitive, we did not introduce the response feedback into its feedback coefficients. This resulted in the sequence of the LFSR being known under a reverse attack. Therefore, we must introduce a second confusion between the LFSR output Q and the APUF to ensure that the APUF challenge is unknown to the attacker. If the required number of authenticated responses is *N*, then *N* responses are generated from the (*n* + 1)th to the (*n* + *N*)th cycle, thereby improving authentication efficiency. During the generation of the final *N*-bit response, the LFSR feedback polynomial is a primitive polynomial, ensuring a complete CRPs space. Next, we will further explain the response generation process through specific examples.

**Figure 7:**
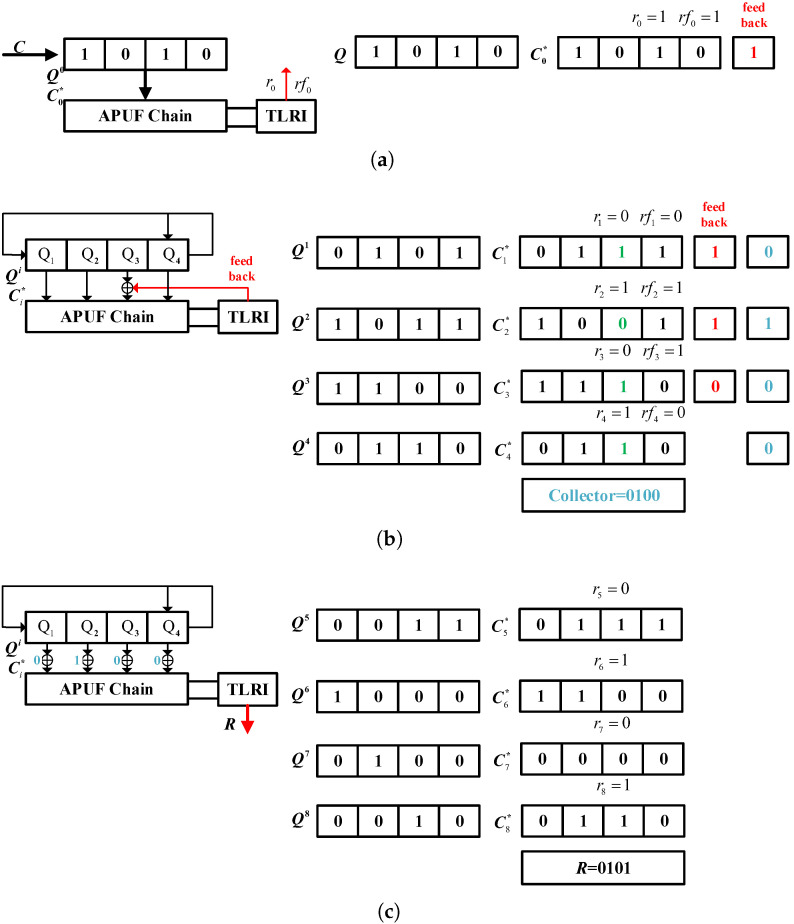
Response generation case: (**a**) initialization; (**b**) stage 1; (**c**) stage 2; rf means reliability flag.

Figure 7 depicts an example of 4-bit APUF response generation, where the feedback polynomial of the LFSR is $f\left(x\right)=x^{4}+x^{3}+1$. The response feedback is located in the third stage of APUF, $Q_{3}$. The red data are the feedback bits, the green data are the result of XOR between the LFSR output and the feedback bits, and the blue data are the bits collected by the collector.

Initialization: Initialize LFSR with challenge *C* = (1010). The initial challenge of APUF $C_{0}^{*}=C$, assuming its response $r_{0}$ = 1 and its reliability flag $rf_{0}$ = 1, indicates that the response will be fed back to the challenge side in the next cycle.

Stage 1: LFSR shifts once and $Q^{1}$ = (0101), XORed with the feedback response $r_{0}$ during the initialization phase. Therefore, the APUF challenge $C_{1}^{*}$ = (0111), assuming its response $r_{1}$ = 0 and its reliability flag $rf_{1}$ = 0. Therefore, the response is not fed back to the challenge side, the feedback response is still 1, and the first bit collected by the collector is 0. In the second cycle, $Q^{1}$ = (1011), XORed with the feedback bit 1, $C_{2}^{*}$ = (1001), assuming $r_{2}$ = 1 and a reliability flag $rf_{2}$ = 1, $r_{2}$ is fed back to the challenge side next cycle, and the second bit collected by the collector is 1. The feedback data are (1110) and the collector data are (0100).

Stage 2: After five shifts, the LFSR state $Q^{5}$ = (0011), and the collector data (0100) are XORed with the output of LFSR to generate an APUF challenge. After four cycles, $C_{5}^{*}$ = (0111), $r_{5}$ = 0, $C_{6}^{*}$ = (1100), $r_{6}$ = 1, $C_{7}^{*}$ = (0000), $r_{7}$ = 0, $C_{8}^{*}$ = (0110), and $r_{8}$ = 1. The final response *R* = (0101).

In conclusion, this structure mainly increases system randomness and nonlinearity by introducing response feedback, which is the root of ensuring resistance to ML modeling and good GSAC properties. TLRI plays a crucial role in improving reliability, as will be analyzed below.

### 4.2. Security Analysis

Traditional approaches to enhancing the security of strong PUFs primarily focus on improving their resistance to ML attacks [42]. In this paper, security is a key focus, not only in terms of resistance to that but also on GSAC, which is important for the resistance to statistical attacks. As can be seen from Section 3, ML modeling attacks often require a PUF mathematical model as a prerequisite to quickly train accurate models. However, when PUF is a black box and the mathematical model is unknown, its attack difficulty will greatly increase. Unlike ML attacks, statistical attackers can exploit the poor GSAC properties of the PUF to directly infer the responses of the remaining challenges from one or more known CRPs without the mathematical model. The ACCA attack scenario proposed in [10] assumes that after receiving the challenges to be attacked, the attacker can selectively choose the remaining challenges and query the PUF to obtain responses, thereby predicting the response of the targeted challenges. Through this method, the PUF responses can be successfully predicted with a probability exceeding 90%. Although this scenario is demanding, it is not unattainable once the attacker gains access to the PUF. Whether it is to enhance resistance to ML modeling or GSAC, increasing the randomness and nonlinearity between challenges and responses is the primary approach. This paper will also analyze the structure proposed in the previous section from this perspective. First, we assume that each response generated by the PUF in stage 1 is sufficiently reliable, which means that all *n* responses will be collected in the collector.

Equation (5) shows the initialization and feedback confusion process in stage 1. $LFSR{\left(C\right)}^{i}$ represents the shifting result of the initial challenge by the LFSR in the *i*-th cycle. APUF() represents the response generated by the APUF after receiving a certain challenge. After receiving the *n*-bit original challenge, the LFSR is initialized, the APUF challenge $C_{0}^{*}$ is *C*, and $r_{0}$ is directly generated. Subsequently, the LFSR shifts and the output result is XORed with $r_{0}$ as the input for the first cycle of the APUF, generating the first response $r_{1}$, which is then stored in the collector. This process is repeated *n* times, i.e., *n* response feedback cycles, and the collector gathers *n* responses.

(5) $$\begin{array}{cc} \; & C_{0}^{*}=C,\;r_{0}=APUF\left(C_{0}^{*}\right)\\ \; & C_{i}^{*}=LFSR{\left(C\right)}^{i}⊕r_{i-1},\;r_{i}=APUF\left(C_{i}^{*}\right),\;i=1,2,\ldots ,n\\ \; & Collector=\left\{r_{1},r_{2},\ldots ,r_{n}\right\} \end{array}$$

Equation (6) shows the response generation process in stage 2, where the response is no longer fed back. To introduce further confusion, the XOR result of the LFSR output sequence and the *n*-bit data in the collector are used as the input challenge for the APUF, generating the required N final responses.

(6) $$\begin{array}{cc} \; & C_{n+j}^{*}=LFSR{\left(C\right)}^{n+j}⊕Collector\\ \; & r_{n+j}=APUF\left(C_{n+j}^{*}\right),\;j=1,2,\ldots ,N\\ \; & R=\left\{r_{n+1},r_{n+2},\ldots ,r_{n+N}\right\} \end{array}$$

As only the initial challenge *C* and the final response *R* are transmitted over an untrusted channel, the attacker cannot obtain direct access to the APUF challenge and response. According to Equation (6), the challenge $C_{i}^{*}$ of APUF in the *i*th cycle is related to the responses of the previous cycles. Therefore, attackers are unable to obtain $C_{i}^{*}$. Due to the random response of APUF, each response will bring 1/2 uncertainty to the system, and the later the generated response, the stronger the nonlinearity. Assuming that all responses are feedback, correctly predicting all the feedback bits requires traversing $2^{n}$ possibilities, which will bring great difficulty to the attacker. After the LFSR shift and the response feedback loop in stage 1, there exists a strong nonlinearity between the challenge *C* and the response R. Even if attackers obtain information such as feedback coefficients through reverse engineering, they still cannot access the data within the collector. Therefore, the PUF model is not a linear relationship expressed by Equations (1) and (3). The attacker cannot directly calculate the APUF feature vector through Equation (3). For GSAC, in addition to the randomness brought about by the feedback process mentioned above, LFSR also plays a significant role. According to the simulation result in Section 5.2, the SAC grows linearly as the flipped bit changes from the 1st to the *n*th bit with the average value of 0.5. When certain bits in the challenge flip, LFSR carries the influence into the generation process of each response in stage 1 through n shifts. Meanwhile, due to the three positions of response feedback dividing the n-stage APUF into four equal parts, there is a 50% probability that the flipping of the previous response will cause the flipping of the next response [10]. In summary, after stage 1, the probability of any challenge bit flipping on response flipping tends to be consistent. This greatly reduces the concept of “most significant” and “least significant” bits [43].

### 4.3. Two-Level Reliability Improvement

From the above analysis, it can be seen that the feedback loop will bring good randomness and nonlinearity, greatly improving the resistance of the PUF to ML modeling and GSAC characteristics. However, since the APUF response is not 100% reliable, each feedback will introduce noise, resulting in low reliability of the final response *R*. According to the reliability simulation results in Section 5.1, in the absence of TLRI, when the underlying APUF reliability is 95.81% the overall output reliability is only 56.29%, approaching an uncontrollable level. This section introduces the specific structure of TLRI, the principle of reliability improvement, and the reduction of MV counts.

**Figure 8:**
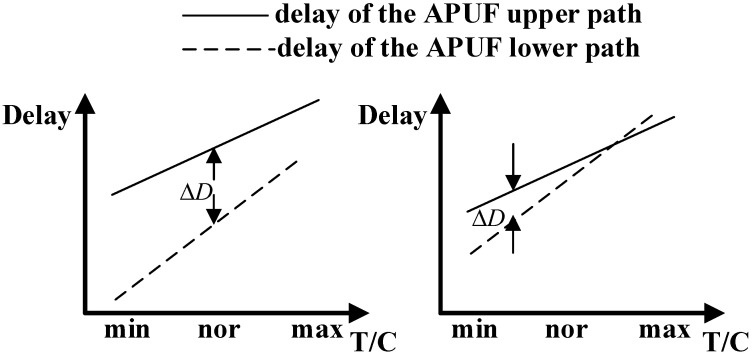
Path delay with different delay difference; the left image represents a large delay difference and the right represents a small delay difference.

The delay difference between the upper and lower paths of APUF follows a normal distribution [41]. As shown in Figure 8, under the influence of noise such as temperature and voltage changes, the positive or negative delay differences of the response with a large delay difference will not change, while the opposite is true for the response with a small delay difference. At the same time, if the delay difference does not meet the setup or hold time of the arbiter, it may lead to metastability, resulting in unreliable responses. Therefore, we can select CRPs with larger delay differences to improve response reliability.

**Figure 9:**
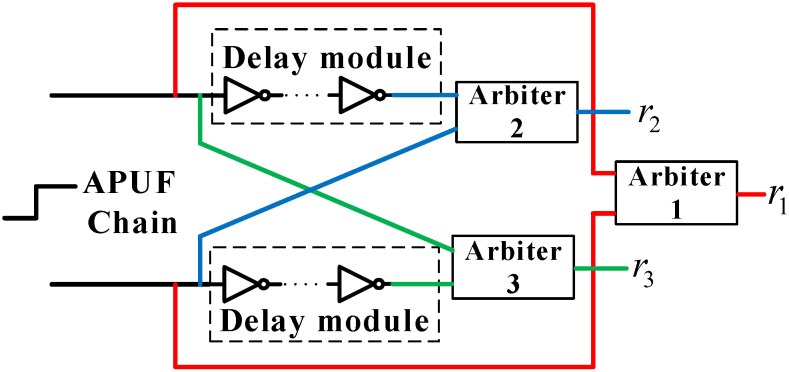
Delay difference test circuit.

To achieve this goal, we modified BST-PUF [39] and constructed a Delay Difference Test Circuit (DDTC) with delay units added at the ends of the upper and lower paths of the original APUF, as shown in Figure 9.

The delay unit is composed of inverters, and the delay size can be changed by changing the number of inverters. Unlike the former, we have added delay units in both the upper and lower paths, which can generate the original APUF response $r_{1}$ and delay detection responses $r_{2}$ and $r_{3}$ in only one cycle. The former requires three cycles to complete the detection, thereby shortening the response generation time. Assuming the delay of the original upper path is D1 and the delay of the lower path is D2, the red link represents the original delay time difference, and the original response $r_{1}$ is generated by Arbiter 1:

(7) $$r_{1}=\mathrm{sgn}\left(D1-D2\right)$$

Assuming the delay unit delay is ΔD, the path delay above blue is $D_{1}+\Delta D$, and the response $r_{2}$ is generated by Arbiter 2:

(8) $$r_{2}=\mathrm{sgn}\left(D1+\Delta D-D2\right)$$

The path delay below green is $D_{2}+\Delta D$, and the response $r_{3}$ is generated by Arbiter 3:

(9) $$r_{3}=\mathrm{sgn}\left(D1-D2-\Delta D\right)$$

If $r_{2}$ is the same as $r_{3}$, it indicates that the original delay difference is greater than ΔD and the reliability is high. According to the results of BST-APUF [39], when the number of inverters in the delay module is more than 4, the error rate of $r_{1}$ filtered out is below $10^{-10}$, which is approximately considered 100% reliable. However, the reliability of $r_{1}$ and $r_{2}$ can also be affected by noise, which can lead to misjudgment of whether $r_{1}$ is reliable, resulting in unreliable feedback. In other words, DDTC transferred the noise from $r_{1}$ to $r_{2}$, $r_{3}$. Therefore, we added a voting circuit after DDTC to improve the reliability of DDTC judgment and the final response *R*.

**Figure 10:**
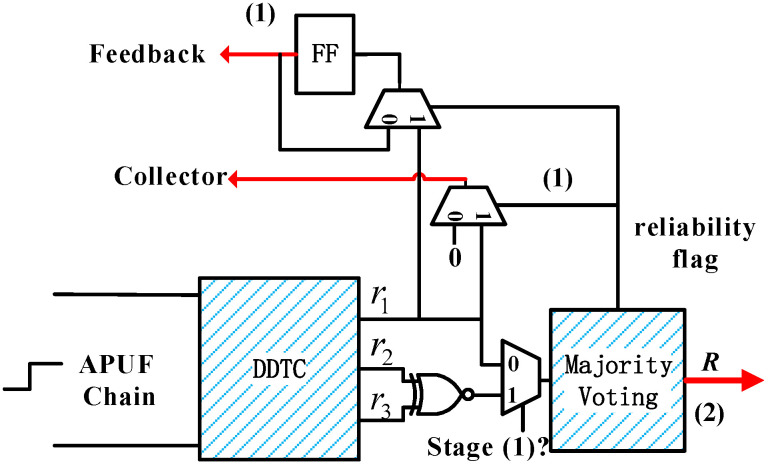
Structure of the two-level reliability improvement.

Figure 10 shows the structure of TLRI, which mainly consists of DDTC and a majority voting circuit. In initialization and stage 1, $r_{2}$ and $r_{3}$ are XNORed and vote on this bit to improve its reliability. If the result is 1, $r_{1}$ will be fed back to the challenge side, otherwise the feedback bit remains unchanged. In stage 2, voting is conducted to improve the reliability of the final response. Similar to [24], we assume that the delay at all stages ($d_{i}^{0/1}$) of APUF obey $N(0,\sigma^{2})$, with additional noise $N(0,\sigma_{noise}^{2})$. From Equation (4), the delay difference of $r_{1}$, $r_{2}$, $r_{3}$ is shown in Equation (10), and additional noise delay $\Delta t_{noise}∼N\left(0,n\sigma_{noise}^{2}\right)$.

(10) $$\begin{array}{cc} \; & \Delta t_{1}=\left(D_{1}-D_{2}\right)∼N\left(0,n\sigma^{2}\right)\\ \; & \Delta t_{2}=\left(D_{1}+\Delta D-D_{2}\right)∼N\left(\Delta D,n\sigma^{2}\right)\\ \; & \Delta t_{3}=\left(D_{1}-D_{2}-\Delta D\right)∼N\left(-\Delta D,n\sigma^{2}\right) \end{array}$$

**Figure 11:**
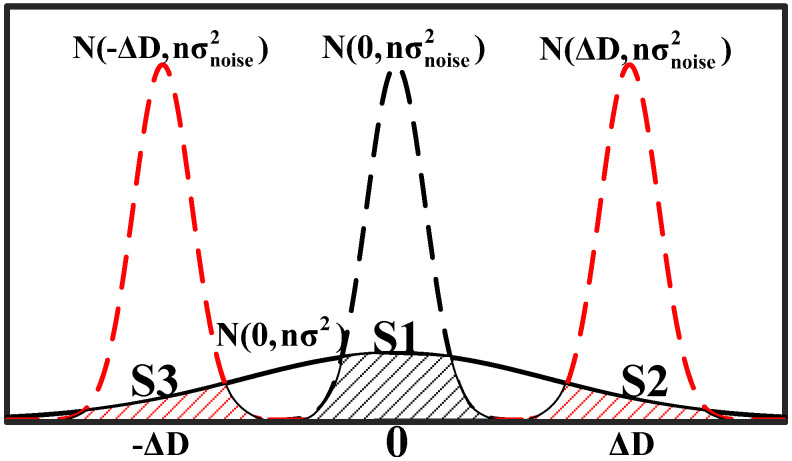
Distribution of delay difference and noise.

To facilitate analysis, we only show the delay difference curve $N(0,n\sigma^{2})$ and shift the noise curve $N(0,n\sigma_{noise}^{2})$ to the left and right, respectively, by ΔD. As shown in Figure 11, we approximate the area where the delay difference distribution curve and the noise distribution curve intersect to analyze the impact of noise on response reliability. S1, S2, and S3, respectively, represent the impact of noise on the responses $r_{1}$, $r_{2}$, and $r_{3}$. The larger the area, the lower its reliability, because the DDTC noise area has changed from S1 to S2 + S3. The larger the delay unit, the smaller the S2 + S3, and fewer votes are required for the voting unit. Meanwhile, as ΔD increases, the number of feedback response bits in stage 1 decreases. However, due to the approximate random distribution of feedback bits among *n* bits, the system’s randomness remains strong. According to the experimental results in Section 5, even with only a few feedback bits, SAC and security are still relatively ideal.

## 5. Numerical Experiments

In this chapter, we explore the various performance aspects of the proposed PUF structure through simulation. In the evaluation of reliability and security, we assume that the APUF has 64 stages and randomly select the LFSR primitive polynomial $f\left(x\right)=x^{64}+x^{4}+x^{3}+x+1$. Based on the Python simulation platform [44], we assume that the standard deviation σ of the delay at all stages, v($d_{i}^{0/1}$), is 1, and the delay of each inverter is 2. From Section 4.3, it can be seen that the larger the DDTC delay, the smaller the average feedback response proportion in stage 1. We take different delays (0, 2, 4, 6, 8, 10, 12) to conduct a relatively comprehensive analysis; 0 represents not using DDTC in stage 1, no response filtering is performed, and all responses are fed back to the challenge side using a pure voting method to improve reliability. The specific feedback ratio is shown in Table 1.

### 5.1. Reliablity Improved with TLRI

In order to ensure a general analysis, we randomly selected 10,000 challenges with different noise standard deviations $\sigma_{noise}=\alpha \sigma (0\leq \alpha \leq 1$), delayed unit delays, and conducted 50 evaluations to calculate their reliability. Consistent with [44], we define PUF reliability as the expected value of its response in the challenge space to evaluate the impact of noise on reliability. Among them, $r^{\left(1\right)}\left(c\right)$ and $r^{\left(2\right)}\left(c\right)$ are the responses of PUF under two identical challenges.

(11) $$Reliability=\underset{c}{E}\;\left(r^{\left(1\right)}\left(c\right)=r^{\left(2\right)}\left(c\right)\right)\times 100\%$$

α is taken as 0.02, 0.05, 0.1, and 0.15, and the corresponding average reliability of the APUF is shown in Table 2. The reliability in FPGA or ASIC is within this range [12,45,46,47].

**Figure 12:**
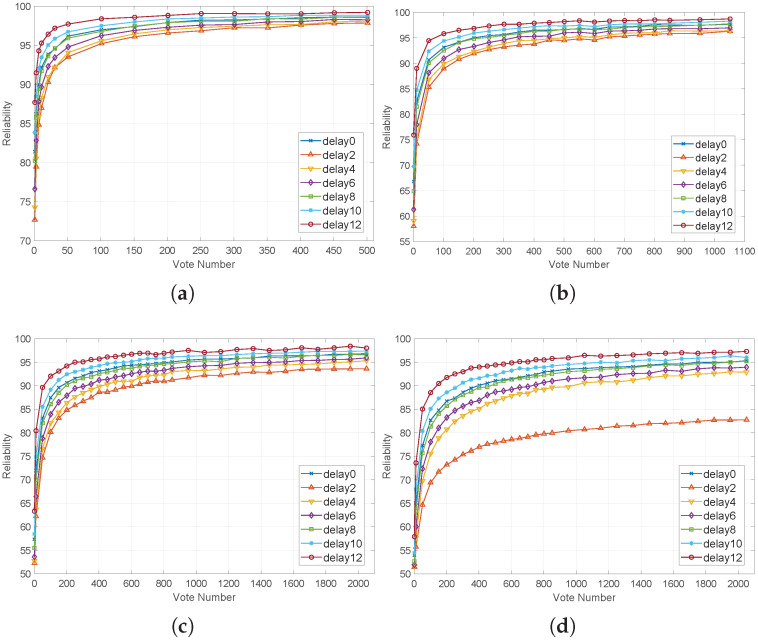
Reliability with different voting numbers: (**a**) α = 0.02; (**b**) α = 0.05; (**c**) α = 0.1; (**d**) α = 0.15.

According to the results in Figure 12, the more votes, the higher the reliability, and the longer the delay, the fewer votes needed to achieve the same reliability. When the delay is 10 or 12 (feedback rate 27.52%, 18.82%), its reliability is higher than that without DDTC under the same number of votes.

In order to quantify the effect of TLRI more intuitively, we calculated the amount of MV required to achieve 95% reliability [37] with a delay of 0, 10, and 12. As shown in Table 3, at different noise levels, the required amount of MV for a delay difference of 10 is 49–70% of the pure voting mode. When the delay is 12, the ratio is approximately 30%. The above results are consistent with the analysis results in Section 4.3. As the delay increases, the noise area decreases and the required amount of MV decreases. When the delay is smaller, the noise area S2 + S3 is larger and a higher amount of MV is required for the same reliability.

As can be seen from Section 4.3, with the help of TLRI the cycle for generating individual response is as follows:

(12) $$Cycles=\frac{(1+n+N)*vot\_num}{N}$$

The total number of cycles includes the number of cycles for initialization, stage 1, and stage 2. Therefore, the ratio of the number of cycles required to generate a single-bit response is consistent with the ratio of the reduction in the amount of voting number.

### 5.2. GSAC

The simulation of GSAC does not consider the impact of noise. Noise increases the randomness of the system, causing an overestimation of GSAC. Regarding the SAC, under different delay units we randomly selected 10,000 challenges and individually flipped a certain bit in the 64-bit challenge. We then calculated the average flip rate of the response when flipping that bit, which serves as the final SAC result.

**Figure 13:**
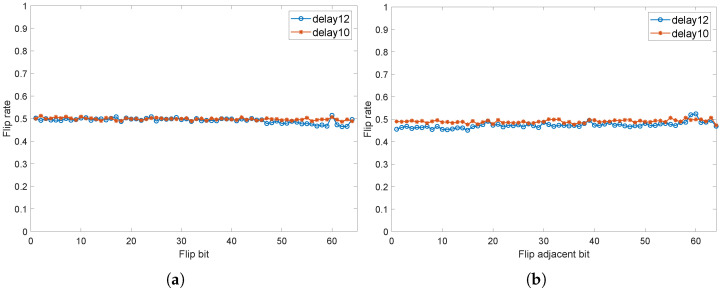
Strict Avalanche Criterion of proposed structure; (**a**) flip single bit, (**b**) flip adjacent bits.

In addition to SAC, we analyzed the avalanche characteristics when flipping two adjacent bits. As stated in Section 5.1, when the DDTC delay is 12 or 10, less MV is required. Therefore, to simplify analysis, this section only considers these two cases. As shown in Figure 13, whether the delay is 12 or 10, flipping a single bit or flipping two adjacent bits results in a response flip rate close to 0.5, indicating good avalanche criterion.

**Figure 14:**
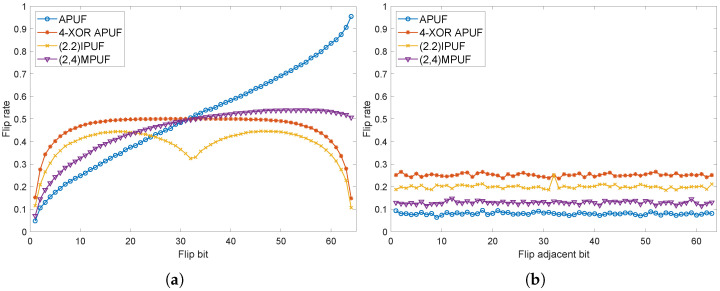
Strict Avalanche Criterion of mainstream PUF; (**a**) flip single bit, (**b**) flip adjacent bits.

Meanwhile, we also simulated the avalanche characteristics of mainstream PUFs. Apart from APUF, the resource consumption of these PUF structures is similar to our proposed structure [25]. As shown in Figure 14, these PUFs do not exhibit ideal statistical characteristics. Especially when flipping the adjacent two bits of the challenge, the response flip rate is far below 0.5. Since these PUFs are all combinations of multiple APUFs, the statistical characteristics will improve as the number of APUFs increases. However, it is difficult to ensure that the response flip rate is around 0.5 for any challenge flipping situation. For example, the 4-XOR APUF shows a significant improvement in its SAC characteristics compared to APUF, but the SAC is still poor when flipping challenges on two sides.

GSAC means that flipping any number of bits in the challenge results in a response flip probability of 0.5. However, simulating all scenarios would incur significant time costs. To simplify the analysis, we randomly selected 100 examples for each number of flipped bits (2, 3, 4, …, 64) to analyze their avalanche criterion. From the above analysis, it is evident that when the delay is 12, the number of votes required for the PUF to achieve 95% reliability is only approximately 30% of the pure voting number. Therefore, we only analyzed the situation with a delay of 12 during the GSAC phase. In fact, as indicated in Section 4.2 and Figure 13, the smaller the delay, the stronger the system’s randomness, resulting in better GSAC. Therefore, if the delay of 12 can satisfy good GSAC, then other situations can also meet the requirements.

**Figure 15:**
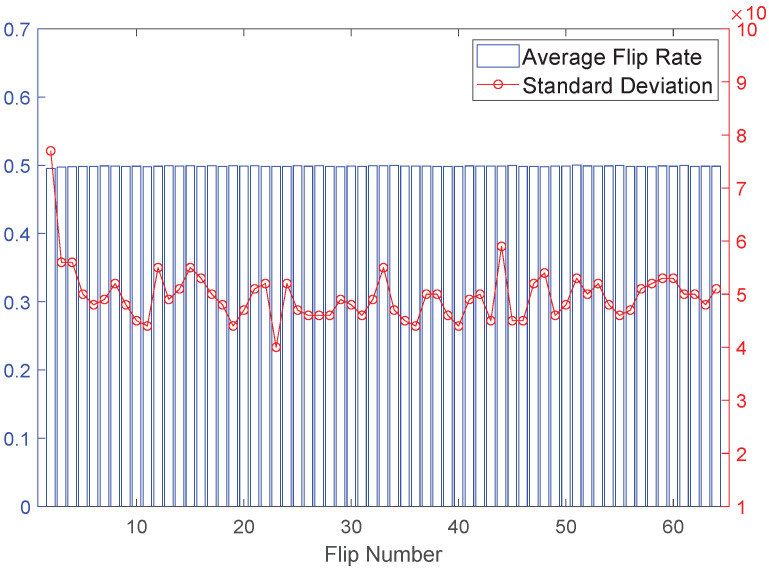
Average flip rate and std of different bit flip numbers.

The experimental results, as shown in Figure 15, indicate that the average response flip probability for 100 examples under any challenge flipping situation is approximately 0.5, with a very small standard deviation fluctuating around 0.005. This result proves that the proposed structure has an ideal GSAC. This means that there is no strong correlation between different CRPs and attackers cannot directly infer the response of one challenge from one or a few CRPs.

### 5.3. Resistance to ML Attacks

This article uses LR, SVM, and DNN to verify the anti-ML ability of the structure. We assume that the attacker can obtain information other than the delay characteristics of APUF itself, including APUF stages, LFSR feedback polynomial, DDTC delay, etc. We collected a total of 1.5 million data as the training set and 10,000 data as the test set.

We use the Python sklearn library to implement LR and SVM attacks, with all parameters set to default values. DNN is built using TensorFlow 2.6.2 and has three hidden layers. The tanh function is used as the activation function for the input and output layers [44]. When the data size is small, a dropout layer is added to prevent overfitting.

**Figure 16:**
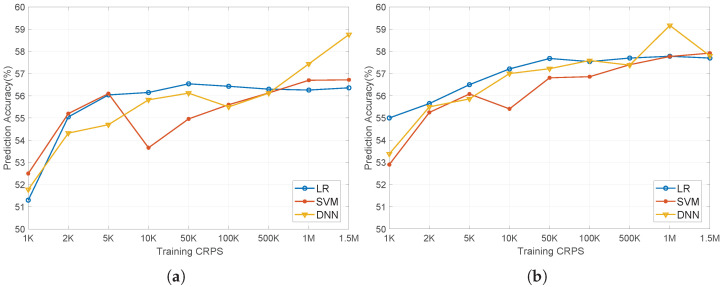
Prediction accuracy of LR, SVM, DNN: (**a**) delay = 10; (**b**) delay = 12.

The results of ML attacks are shown in Figure 16. Even if the training data reach 1.5 million CRPs, the prediction rate of the three ML mathods will not exceed 60%.

Although the feedback coefficients of the LFSR itself are not obfuscated, its output can be inferred when generating the final response in stage 2. However, due to the XOR operation in the collector, the final challenge becomes unpredictable. The data in the collector consist of response bits stabilized in stage 1, and thanks to the 64-stage cyclic feedback it possesses strong nonlinearity and unpredictability. Therefore, both LR and SVM, commonly used for linear prediction, and DNN, used for non-linear modeling, find it challenging to achieve high prediction rates.

In summary, when the delay of the DDTC module is set to 10 (response feedback rate 27.52%) or 12 (response feedback rate 18.82%) the proposed PUF can satisfy GSAC and resist ML modeling attacks with fewer MV cycles to achieve 95% reliability.

### 5.4. Uniformity and Uniqueness

Uniformity refers to the distribution of the 0/1 of PUF responses. In an ideal scenario, the proportion of 0/1 in the response generated by the same PUF under different challenges should be 50%, which is the basis for the good randomness of PUF. It is usually defined by Hamming distance.

(13) $${\left(Uniformity\right)}_{i}=\frac{1}{n}\sum_{l=1}^{n}r_{i,l}\times 100\%$$

$r_{i,l}$ is the *l*-th response of the *i*-th PUF instance among n responses. We evaluated the Uniformity of 100 PUFs, each using 1000 challenges to generate 1000 pairs of 64-bit responses.

Uniqueness refers to the differences in responses between different PUF instances, which is typically represented by the inter-chip Hamming distance.

(14) $$HD\left(R_{i},R_{j}\right)=\sum_{n=1}^{N}\left(R_{i}\left[n\right]⊕R_{j}\left[n\right]\right)$$

$R_{i}$ and $R_{j}$ represent the responses generated by different PUF instances under the same challenges and N represents the number of PUF response bits. The uniqueness is defined as follows for K PUF instances:

(15) $$Uniqueness=\frac{2}{K(K-1)}\sum_{i=1}^{N-1}\sum_{j=i+1}^{N}\frac{HD(R_{i},R_{j})}{N}\times 100\%$$

In this paper, taking *K* as 10, the inter-chip Hamming distance between two PUF instances is also measured using 1000 sets of 64-bit responses.

The Uniformity and Uniqueness are shown in Table 4. Regardless of whether the APUF has 32, 64, or 128 stages, the Uniformity and Uniqueness are close to the ideal value of 50%. Since the structure of the APUF itself has not been modified, the delay size does not have a significant impact on these two characteristics.

## 6. Conclusions

Security and reliability have always been the focus of attention in the PUF field. However, improving security often accompanies a more complex structure, introduces more noise, and results in unreliable responses. In this paper, security not only includes the ability to resist ML modeling attacks but also encompasses the ability to resist statistical attacks. Based on LFSR and APUF, this paper constructs a response cyclic feedback PUF. The GSAC of this structure fluctuates around 0.5, and the success rate of LR, SVM, and DNN ML modeling attacks does not exceed 60%. At the same time, we have proposed a two-level reliability improvement method, which can reduce the number of votes and the generation time of single-bit response by more than 65% while ensuring good security, achieving a balance between security and reliability at a relatively reasonable cost. 

## Figures and Tables

**Table 1:** Feedback rate (%) of different delays.

**Delay**	0	2	4	6	8	10	12
**Feedback rate (%)**	100	82.88	66.53	51.41	38.51	27.52	18.82

**Table 2:** Average reliability (%) of APUF with different noise.

α	0.02	0.05	0.1	0.15
**Reliability (%)**	99.15	97.88	95.81	93.72

**Table 3:** Vote number (percentage of no DDTC) required for 95% reliability with different delay and noise.

α	0.02	0.05	0.1	0.15
**Delay**				
0	35	215	751	1649
10	17 (49%)	119 (55%)	467 (62%)	1151 (70%)
12	9 (26%)	61 (28%)	251 (33%)	499 (30%)

**Table 4:** Average (%) and Standard Deviation (%) of Uniformity and Uniqueness for simulated PUF.

*n*	Delay	Uniformity (Avg,Std)	Uniqueness (Avg,Std)
32	10	49.54 (3.67)	50.17 (1.41)
	12	49.56 (3.68)	50.24 (3.54)
64	10	50.05 (2.79)	50.00 (0.31)
	12	50.04 (2.83)	50.03 (0.28)
128	10	49.93 (1.78)	49.99 (0.21)
	12	49.87 (1.78)	50.04 (0.17)

## Data Availability

Data are contained within the article.
